# Gut microbiota influences feeding behavior via changes in olfactory receptor gene expression in Colorado potato beetles

**DOI:** 10.3389/fmicb.2023.1197700

**Published:** 2023-06-28

**Authors:** Hongwei Li, Yanxue Yu, Jian Zhang, Yuhan Wang, Liu Zhang, Junfeng Zhai, Yongjiang Zhang

**Affiliations:** ^1^Institute of Plant Quarantine, Chinese Academy of Inspection and Quarantine, Beijing, China; ^2^CAIQ Center for Biosafety in Sanya, Sanya, China; ^3^Technology Center of Suifenhe Customs District, Mudanjiang, China; ^4^Biowavelet Ltd., Chongqing, China

**Keywords:** gastrointestinal microbiome, anti-bacterial agents, odorant receptor, *Pseudomonas*, feeding behavior

## Abstract

The Colorado potato beetle (CPB) is an internationally recognized plant quarantine pest that causes serious losses to potato agricultural production. The gut microbiota plays an important role in its growth and development, and the olfactory system plays an important role in insect feeding behavior. The gut microbiota is known to be capable of inducing changes in the olfactory systems of insects. However, the way these associated gut microbes influence the feeding-related behaviors of CPBs remains unclear. To explore the relationship between them, fresh potato leaves immersed in a mixture of five antibiotics (tetracycline, penicillin, ofloxacin, ciprofloxacin, and ampicillin) at specific concentrations for 1 h were fed to adult CPBs to reduce the abundance of gut microbes. We found that the feeding behavior of CPBs was significantly affected by the gut microbiota and that *Pseudomonas* was significantly higher in abundance in the control group than in the antibiotic group. We then used transcriptome sequencing to explore the differences in olfactory receptor genes in the heads of non-treatment and antibiotic-fed CPBs. Through Illumina Hiseq™ sequencing and screening of differential genes, we found that the olfactory receptor gene LdecOR9 was significantly upregulated and LdecOR17 was significantly downregulated after antibiotic feeding. A real-time polymerase chain reaction was used to verify the changes in olfactory receptor gene expression in the non-treatment groups and antibiotic-treated groups. The feeding behavior was partially rescued after CPBs were re-fed with intestinal bacteria. These results indicate that a certain amount of gut microbiota can result in the loss of the olfactory discrimination ability of CPBs to host plants. In summary, this study investigated the relationship between gut microbiota and olfactory genes, providing a reference for research on microbial control.

## 1. Introduction

A wide variety of microorganisms colonize the digestive tract of insects in the form of gut microbial communities. These symbiotic bacteria participate in the speciation and most life activities of insects and play an important role in many physiological functions, such as growth and development, reproduction, nutrient metabolism, immunity, pesticide resistance, and host communication (Crotti et al., 2012). In addition, they help the host insect avoid the invasion of predators, parasites, and pathogens, and directly or indirectly affect the physiology and health of insects. Intestinal microorganisms can regulate host appetite by influencing the host’s central nervous system (Sampson and Mazmanian, 2015). Based on the important functions of gut microbes and host insects, the study of gut microbes can provide new ideas for green pest control.

In recent years, there has been an increasing number of studies on intestinal microbes in different countries, with the research scope becoming increasingly extensive. After long-term evolution, a large number of microorganisms in the insect gut are in a microenvironment of dynamic balance, the stability of which plays an important role in the growth and development of the insect. Once the dynamic balance of this environment is disrupted, the physiological functions of host insects become disturbed, thus exhibiting an abnormal state. Symbiosis provides an opportunity for gut microbiota to live together through reciprocal or unidirectional benefits with insects, and it regulates insect behavior by affecting their olfactory function (Jefferis et al., 2007; Masuda-Nakagawa et al., 2009; Slankster et al., 2019; de Paula et al., 2021; Ai et al., 2022). Gut *Lactobacillus* strains in honeybees can change tryptophan to indole derivatives that stimulate the host aryl hydrocarbon olfactory receptor (OR), thereby promoting memory behaviors. Gut bacteria may largely influence insect olfaction by affecting neural circuit communication (Zhang et al., 2022). In *Drosophila*, microbiologically sterile flies display a moderate reduction in memory tests during olfactory appetitive conditioning (Silva et al., 2021). The gut microbiota of *Drosophila* larvae was found to be altered by treatment with antibiotics or probiotics, and the tropism response of the larvae treated to odorants was reduced (Wilson and Laurent, 2005; Slankster et al., 2019). *Wolbachia*-infected *Drosophila* (fruit fly) might increase sensitivity towards food odors by having much higher transcript levels of the OR gene. The OR co-receptor in the fruit fly was found to be hyperactive under sterile or antibiotic-treated conditions, indicating that the symbiotic bacteria in the gut of the insect greatly influence its behavior (Peng and Wang, 2009). These findings provide a good opportunity for further research on the relationship between commensal bacteria and their hosts.

Although intestinal microbes in *Leptinotarsa decemlineata* (Colorado potato beetle; CPB) have been reported (Yu et al., 2021), the relationship between intestinal microbes and OR genes remains to be studied. This study aimed to investigate the relationship between intestinal microbes and olfactory genes. Adult CPBs were fed a compound antibiotic solution consisting of tetracycline, ciprofloxacin, penicillin, ofloxacin, and ampicillin, and the effects of gut microbiota on the olfactory behavior of CPBs were investigated using an insect olfactory analyzer. The findings may provide basic data for exploring the mechanism of intestinal symbiotic bacteria and insect behavior and for the future development and utilization of new green prevention and control methods. Furthermore, this study provides a reference for research on the olfactory behavior of CPBs and their biological control agents.

## 2. Materials and methods

### 2.1. Insects

Healthy adult CPBs were collected from Suifenhe, Heilongjiang Province, and cultured with fresh potato leaves in the Safety Laboratory of Suifenhe Customs Technology Center, Heilongjiang Province. The temperature of the greenhouse was 26 ± 2°C, the relative humidity was 40–60%, and the photoperiod was 16 L:8D.

### 2.2. Effects of gut microbiota treatment on the feeding selection behavior of CPBs

Insect feeding behavior is initiated by the volatiles emanating from the leaves of a host plant, and it is primarily governed by olfaction. To explore the factors influencing feeding selection behavior in CPBs, fresh potato leaves were immersed in a mixture of five antibiotics (tetracycline, penicillin, ofloxacin, ciprofloxacin, and ampicillin) at specific concentrations (50 μg/mL, 100 μg/mL, and 1 mg/mL) for 1 h. The five antibiotics in the mixture were mixed at the same ratio, and 1% Tween-20 was added to improve the adsorption capacity of the antibiotics on potato leaves. The experimental potato beetles were fed leaves soaked in each concentration of antibiotic mixture for 3 days, and the soaked potato leaves were replaced once a day. The control group was fed sterile water and potato leaves soaked in 1% Tween-20 for 3 days, and the development and feeding conditions were the same to eliminate the influence of other factors on their feeding selection behavior. The feeding of the experimental and control group was stopped 6 h before the test. The insect olfactory behavior instrument was divided into treatment and control areas. Fresh potato leaves (30 g) were placed in the treatment area, and the control area was blank. Each test time was 30 min, and the number of selected insects on each wall was recorded after the test time. Each experiment was repeated three times. We maintained a constant light intensity in the experimental area. The attraction index was calculated as the treatment group minus the control group divided by the total.

### 2.3. 16S rRNA sequencing, transcriptomic, and gene expression analyses

For 16S sequencing of the gut microbes of antibiotic-treated and non-treatment control CPBs, genomic DNA was extracted using the Fast DNA Stool Mini Kit (Qiagen, Germany). The quality and quantity of the extracted DNA were measured on a 1% agarose gel and using a Nanodrop, respectively. This DNA was used as a template for amplifying 16S V3–V4 fragments by RT-PCR using primers 341F (5′-CCTAYGGGRBGCASCAG-3′) and 806R (5′-GGACTACH VGGGTWTCAAT-3′) (Frank et al., 2013). The libraries were constructed using amplified PCR products, which were then sequenced using an Ion Torrent S5TM XL platform.

The PureLink™ RNA Mini Kit was used to extract RNA from whole heads of CPBs in the antibiotic treatment and control group, and the Illumina Hiseq™ sequencing platform was used for RNA transcriptome sequencing. Expression levels were assessed in terms of TPM values (transcripts per kilobase per million reads), which were calculated based on the number of mapped transcript fragments corrected for transcript length and sequencing depth through featureCounts (http://subread.sourceforge.net) software. Differential expression analysis of two samples was performed using the DESeq2 R package (Love et al., 2014). The software applied the negative binomial generalized linear regression model to fit gene expression, evaluate the variation of dispersion and difference multiple, and Wald test was used for statistical test to obtain *p* value. Differentially expressed gene screening requirements were|log2fold change| > 1, and a *p*-value <0.05 (Trapnell et al., 2010).

The qRT-PCR was performed in a 20-μL reaction mixture (including 1 μL of cDNA, 10 μL of 2× TransStart®Green qPCR SuperMix, 1 μL of 10 μM forward and reverse primers, and 7 μL of nuclease-free water) on an ABI 7900 system (Applied Biosystems, CA, United States) to compare the differences in the expression of OR genes between the antibiotic treatment, control, and Rescue group in the heads of CPBs. It was performed under the following conditions: 95°C for 2.5 min and 36 cycles at 94°C for 15 s, 60°C for 15 s, and extension at 72°C for 15 s. The primers (5′-3′) used were as follows: OR9 forward primer, CTTTTCTAGGAGCCCAAC; OR9 reverse primer, ATTACTCTTGAAAGGCCAT; OR17 forward primer, ATAGTATTATGACTAATAGCGAAG; and OR17 reverse primer, GAAAATTATGGGCGCTTT. The melting curve was analyzed to assure the specificity of the primers after each reaction, and the RP18 gene was used as the reference gene (Shi et al., 2013). The 2^−ΔΔCT^ method (Livak and Schmittgen, 2001) was used to calculate the expression level of each OR gene. Each sample type was repeated three times. The differences between the relative expression levels of OR genes were analyzed using a *t*-test. The experimental data were statistically analyzed using GraphPad Prism 5 (GraphPad Software, CA, United States).

### 2.4. Effects of feeding target microorganisms on the feeding behavior of antibiotic-treated adult CPBs

We used traditional microbial separation and purification techniques to isolate the intestinal tracts of adult CPBs from Heilongjiang province. We obtained different monoclonal strains and cloned and sequenced 16S rDNA for each. We cultured three *Pseudomonas* strains (*P. rhodesiae* 39B5, *P. rhodesiae* VTT E-031889, and *P. plecoglossicida* sp. RL104) separately and diluted the concentration of the bacterial solution to 10^5^ cells/mL. To prepare the recovery group for experiments, we mixed 50 mL of each of the above three bacterial solutions, soaked fresh potato leaves in 150 mL of the bacterial mix solution for 1 h, and then fed to CPB adults after 3 days of antibiotic treatment (1 mg/mL). The feeding process was repeated for 3 consecutive days. We compared the feeding choice behavior among the three groups, which were the Rescue group and two control groups (one treated with antibiotics and one treated with sterile water using the method in section “Effects of gut microbiota treatment on the feeding selection behavior of CPBs”). The methods of behavior testing and attraction index calculation were the same as those described in section “Effects of gut microbiota treatment on the feeding selection behavior of CPBs”.

## 3. Results

### 3.1. Feeding behavior changed after treatment with antibiotics

**Figure 1:**
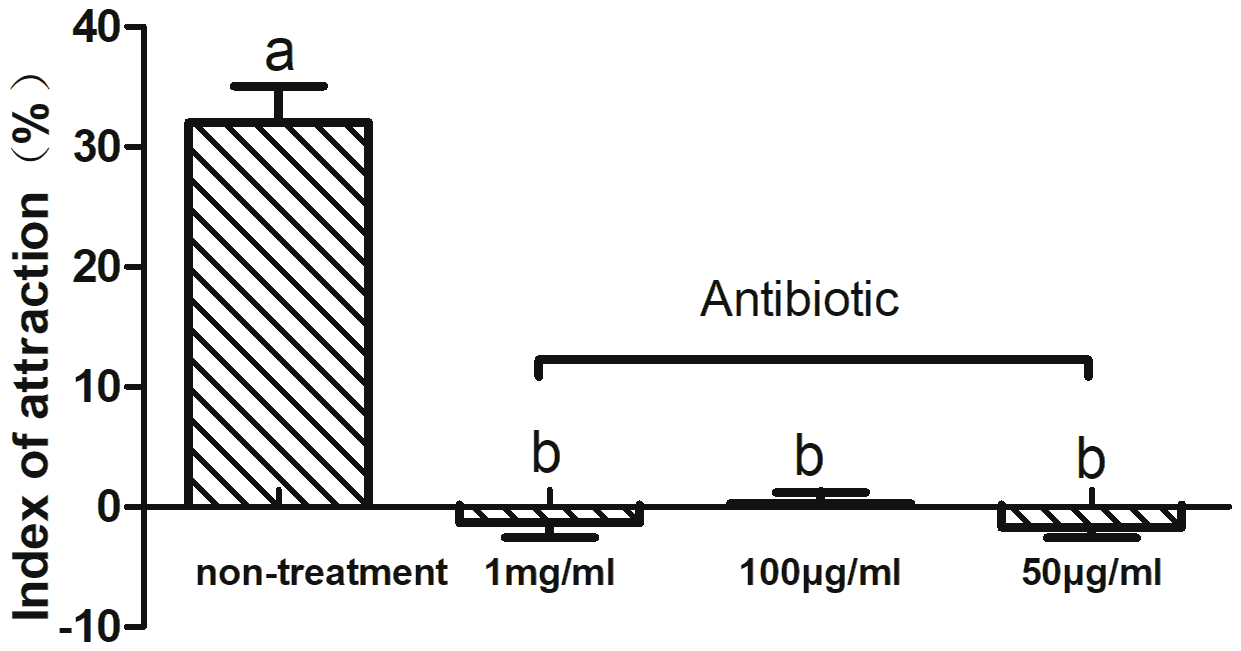
Feeding behavior changed after treatment with antibiotics. Adult CPBs fed antibiotic-treated leaves (50 μg/mL, 100 μg/mL, and 1 mg/mL) and clean leaves (non-treatment).

To investigate the relationship between intestinal microbes and olfactory-mediated feeding behavior in CPBs, we fed adult CPBs with a compound antibiotic solution consisting of tetracycline, ciprofloxacin, penicillin, ofloxacin, and ampicillin to alter the abundance of microbes. The effects of antibiotics on the olfactory behavior of CPBs were investigated via insect olfactory analysis. The different effects of the mixed antibiotic solution on the leaf selection behavior of CPBs were analyzed. The feeding behavior of CPBs was significantly changed by feeding on the leaves soaked in the antibiotic mixture, and the selection of host plant leaves was significantly reduced. After feeding with antibiotics, CPBs lost their ability to distinguish between leaves and blank controls (the attraction index was close to zero). We selected different concentrations of antibiotics for feeding treatment, including 1 mg/mL, 100 μg/mL, and 50 μg/mL. No significant difference was observed in the selection of host plant leaves by CPBs with different concentrations of antibiotics (Figure 1). These results indicate that a dose of 50 μg/mL of antibiotics may be sufficient to reduce the olfactory discrimination ability of CPBs to host plants, which can be developed and used as a new green control method in the future.

**Figure 2:**
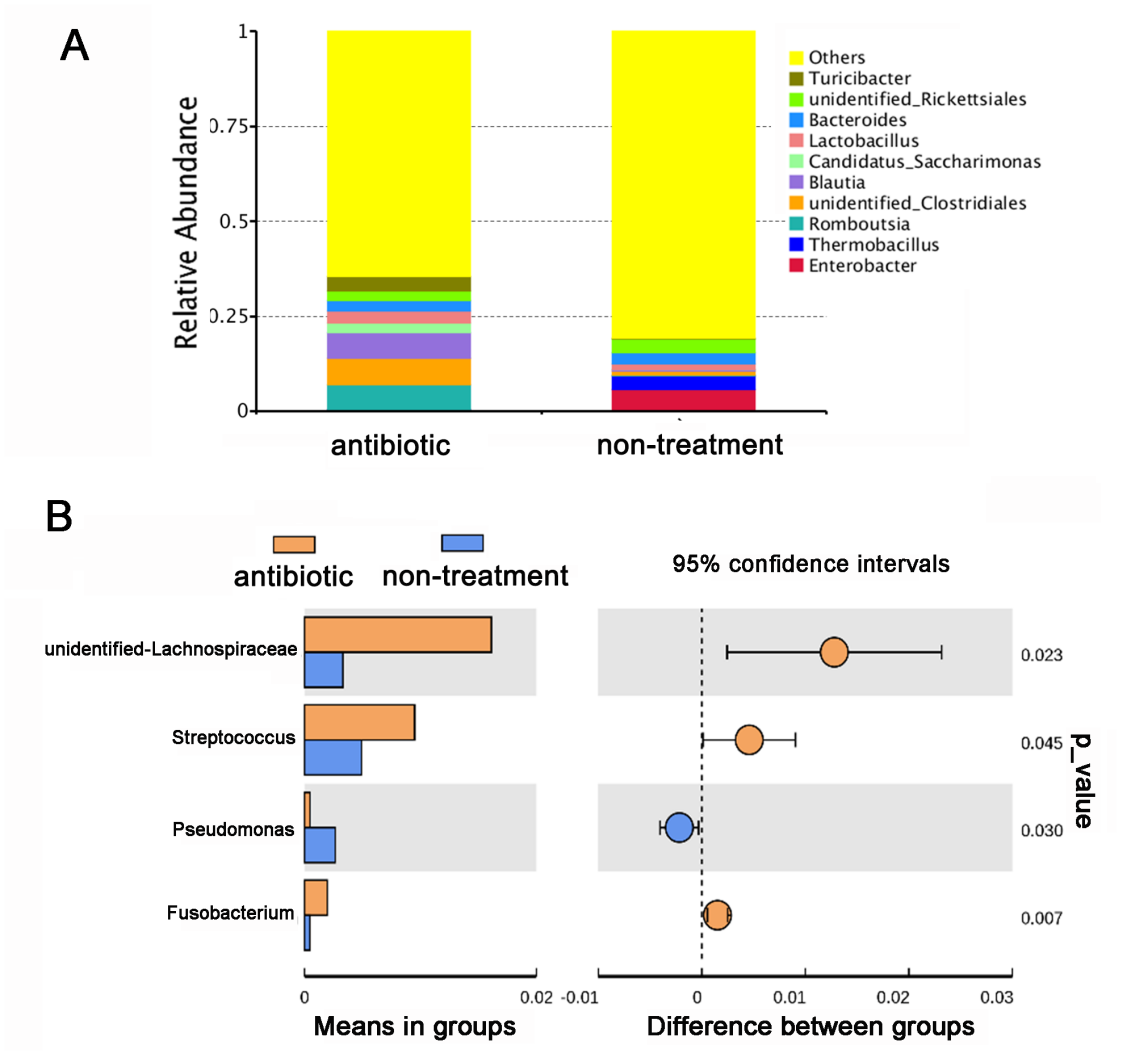
Antibiotics alter the gut microbial community of *Leptinotarsa decemlineata*. **(A)** Taxonomic composition of gut microbiota in the antibiotic and non-treatment groups at the genus level based on the V3–V4 region of the 16S rDNA gene sequencing. **(B)** Comparison of the relative abundance of different microorganisms at the genus level using a *t*-test.

Based on the changes in the olfactory behavior of adult CPBs after feeding with a mixture of antibiotics, we performed 16S sequencing of intestinal microorganisms in adult CPBs treated with 1 mg/mL antibiotics and in non-treatment control CPBs to explore the differences in the composition of intestinal microorganisms between the two groups. A total of 46 phyla, 65 classes, 154 orders, 286 families, 779 genera, and 951 species were obtained from the intestinal microorganisms of the CPBs in the two groups. The number of annotations obtained for the two groups at different taxonomic levels is listed in Table 1. The results showed that the gut microbiota diversity of the control group was slightly higher than that of the antibiotic group, and the relative abundance of bacteria between the two treatments differed significantly at each classification level (Figure 2). The relative abundance of *Pseudomonas* was significantly higher in the control group than that in the antibiotic group (*p* = 0.030).

**Table 1:** Summary data of gut microbiota composition in CPBs under different treatments at various taxonomic levels.

Sample	Phylum	Class	Order	Family	Genus	Species
Antibiotic	33	56	109	226	552	604
Non-treatment	42	56	134	255	644	650
Total	46	65	154	286	779	951

Non-treatment represents CPBs fed with only fresh potato leaves. Antibiotic represents CPBs fed with fresh potato leaves treated with antibiotics.

### 3.2. Changes in OR gene expression after antibiotic feeding

**Figure 3:**
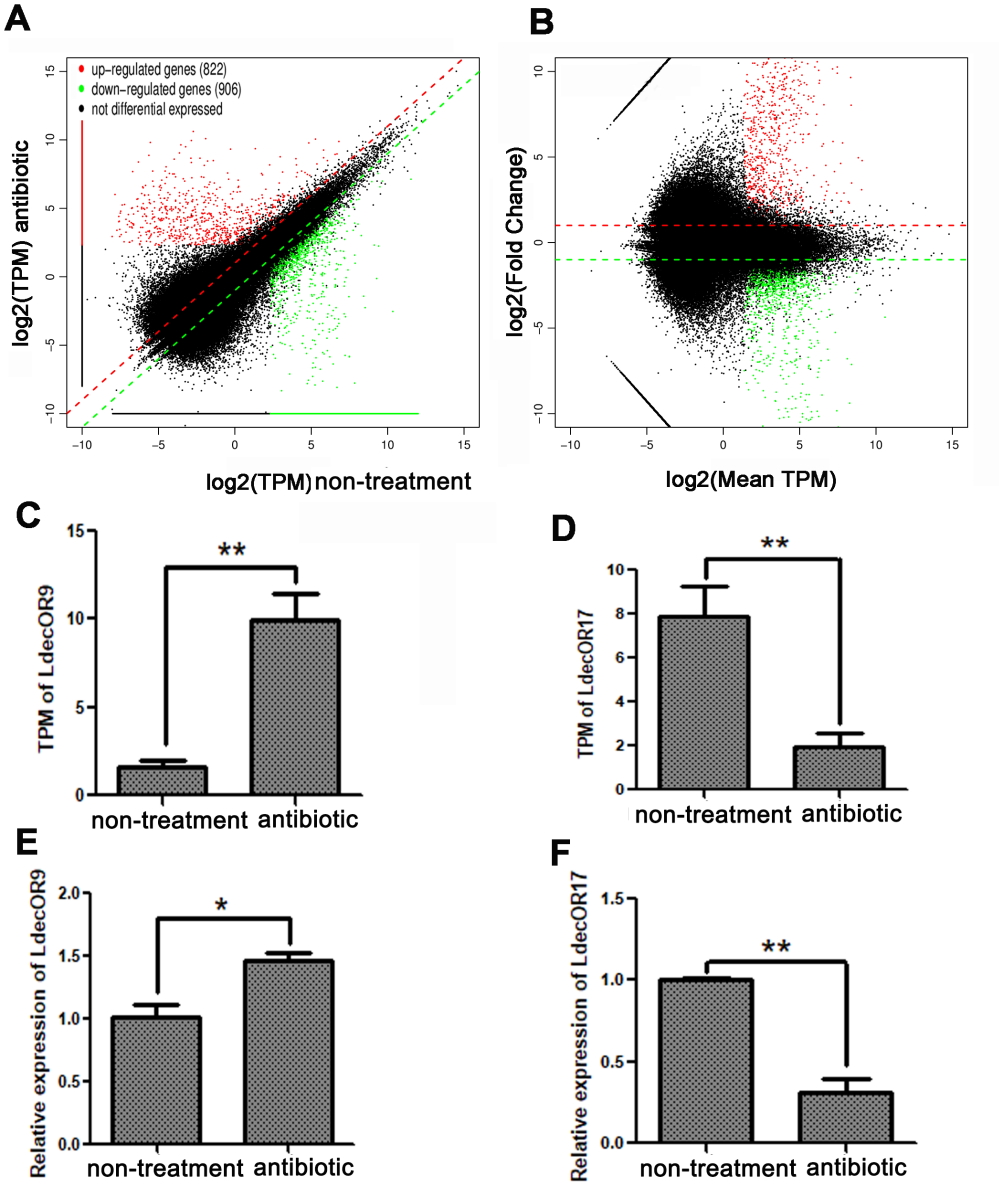
Changes in OR gene expression in *L. decemlineata* after antibiotic feeding. **(A,B)** Visualization of transcripts with significant differential expression. (**C**) Transcripts per million (TPM) value of LdecOR9. **(D)** TPM of LdecOR17. **(E)** Change in the relative quantitative expression level of LdecOR9 after antibiotic feeding determined by qPCR. **(F)** Change in the relative quantitative expression level of LdecOR17 after antibiotic feeding determined by qPCR. Non-treatment, CPBs fed with only fresh potato leaves; Antibiotic, CPBs fed with fresh potato leaves treated with antibiotics.

To investigate the changes in OR gene expression induced by gut microbiota, we screened the expression differences of all genes through transcriptome analysis with a reference genome. According to the results of all samples and splicing, the transcript per million (TPM) value was used to screen differentially expressed genes. Compared with the non-treatment group, 1728 significantly differentially expressed genes in the three antibiotic treatment groups were identified, of which 822 were upregulated and 906 were downregulated. According to the mean TPM value ≥5, |log_2_fold change| > 1, and a *p*-value <0.05, only one upregulated OR gene, *LdecOR9*, and one downregulated OR gene, *LdecO17*, were identified (Figures 3A,B). The detailed data can be found in Supplementary Data 1. The OR genes *LdecOR9* and *LdecOR17*, with significant differences, were validated via quantitative real-time fluorescence PCR, with the *RP18* gene as the internal reference gene. Comparing the results of qPCR with the TPM results of transcriptome sequencing, the expression trends of *LdecOR9* and *LdecOR17* were consistent (Figures 3C–F), which further indicates that the transcriptome sequencing results were reliable.

### 3.3. Feeding behavior was partially rescued after CPBs were re-Fed with *Pseudomonas*

The use of antibiotics has a great impact on gut microbiota; thus, we adopted a feeding method to restore intestinal gut microbiota in CPBs. We then compared the changes in CPB feeding behavior in three strains of *Pseudomonas* (*P. rhodesiae* 39B5, *P. rhodesiae* VTT E-031889, and *P. plecoglossicida* sp. RL104; bacterial concentration of 10^5^). These strains were isolated from the intestinal tract of adult CPBs from Heilongjiang province and were chosen based on previous 16S rDNA sequencing analysis results and the literature review. After feeding antibiotic-treated adult CPBs, which had marked changes in feeding selection behavior, we used the *Pseudomonas* strains to verify the behavior experiments and investigate the effects of gut microbiota on the olfactory behavior and olfactory-related genes in adult CPBs.

**Figure 4:**
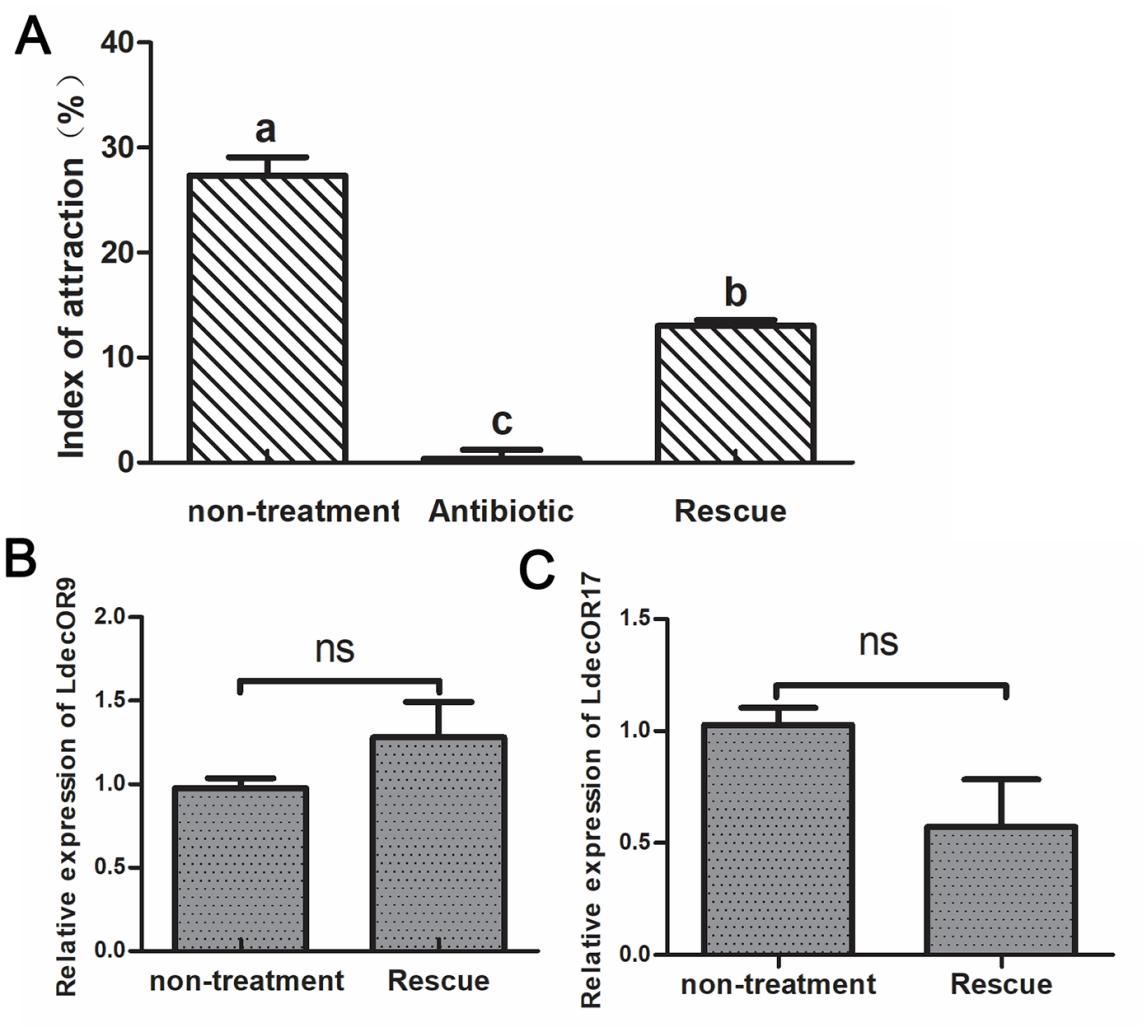
Feeding behavior was partially rescued after CPBs were re-fed with intestinal gut microbiota. **(A)** Comparison of the index of attraction among the different groups. **(B)** Comparison of the relative quantitative expression level of LdecOR9 between non-treatment and Rescue. **(C)** Comparison of the relative quantitative expression level of LdecOR17 between non-treatment and Rescue. Non-treatment, CPBs fed with only fresh potato leaves; Antibiotic, CPBs fed with fresh potato leaves treated with antibiotics; Rescue, CPBs treated with antibiotics and re-fed with intestinal bacteria.

The fresh potato leaves were soaked in the *Pseudomonas* bacterial mixture at a concentration of 10^5^ to feed the antibiotic-treated adult CPBs. We then observed whether their feeding choice behavior was restored. As can be seen from the results, the index of attraction to host plant leaves was approximately 28% in the non-treatment group and approximately 0% in CPBs fed with leaves soaked in a 1 mg/mL antibiotic mixture, which shows changes in feeding selection behavior. The feeding selection behavior of adult CPBs was significantly recovered, reaching approximately 15%. The results showed that antibiotic feeding had a significant effect on the olfactory behavior of adult CPBs. The effects of antibiotics could be partially recovered, but incomplete recovery may be mainly due to the complex gut microbial population. Feeding only the above target bacteria could not completely recover the bacterial population dynamics in CPBs. We further verified the two olfactory genes *LdecOR9* and *LdecOR17* using quantitative real-time PCR and found that gene expression was partially restored (Figure 4), which was correlated with the changes in intestinal microorganisms, further proving that gut microbiota influence feeding behavior through changes in OR gene expression in CPBs.

## 4. Discussion

Antibiotics have been widely used in experiments as agents that can rapidly and effectively affect the gut microbiota of insects (Cho et al., 2012; Chu et al., 2013). In this study, we found that after CPBs were fed antibiotics, their feeding behavior was influenced by significant changes in the expression abundance of the gut microbiota. To explore the differences in OR gene expression in the heads of non-treatment and antibiotic-treated CPBs, we performed transcriptome sequencing and qPCR and found that the expression of some OR genes significantly changed after antibiotic feeding. Feeding behavior was partially rescued after CPBs were re-fed with intestinal bacteria, and the target OR gene expression level was restored. Based on the results, it appears that gut microbiota could greatly influence insect olfaction by affecting neural circuit communication.

The phenomenon of gut microbes influencing insect olfactory behavior has been reported. The gut microorganisms of *Blattella germanica* (German cockroach) contribute to the production of chemical pheromones, including volatile carboxylic acids, which can effectively regulate the aggregation agent in their feces, thus inducing the aggregation behavior of German cockroaches (Ayako et al., 2015). Gut microbiota alters olfactory-guided microbial preferences and foraging decisions in fruit flies (Wong et al., 2018). *D. melanogaster* can be treated with *Lactobacillus brevis*, a symbiotic gut bacterium that can improve its walking speed and daily activities (Schretter et al., 2018). Essential amino acids and their intestinal symbiotic bacteria, *Acetobacter* and *Lactobacillu*s, can synergistically affect the food selection of *Drosophila* (Leitão-Gonçalves et al., 2017). *Erwinia carotovora carotovora*-infected flies showed enhanced selection abilities for 4-methylcyclohexanol and 3-octanol compared with the control group (Shih et al., 2019). Larvae treated with antibiotics had a reduced microbiota load and exhibited a reduced chemotaxis response towards odorants compared to the control animals. This behavioral phenotype was partially rescued in larvae treated with probiotics, which resulted in the partial recovery of microbiota loads (Qiao et al., 2019). These microbial preferences are largely olfactory guided and have a profound impact on host foraging. The present study also showed a certain relationship between the intestinal microbes of CPBs and olfactory behavior. However, the mechanism by which intestinal microbes affect feeding behavior remains unclear, and the pathway through which intestinal microbes alter the OR genes of CPBs to affect feeding warrants further study. In future research, it is necessary to specify the influence of several intestinal microorganisms and the relationship between gut microbial communities and olfactory genes, such as the influence of *OR9* and *OR17* on the feeding behavior of CPBs, to explore the olfactory behavior mechanism of intestinal symbiotic bacteria and insects.

In summary, the expression of olfactory genes was examined by feeding CPBs with antibiotics, providing basic resources for gene screening for biological control and references for further research on the molecular mechanisms related to the olfactory behavior of CPBs. The study of the relationship between intestinal microbes and host behaviors has greatly enriched our understanding of the role of intestinal microbes in insects and, to a certain extent, uncovered the effect of intestinal microbes on host behaviors.

## Data availability statement

The data presented in this study have been deposited to the NCBI Gene Expression Omnibus (GEO), accession number GSE235447.

## Author contributions

HL, YY, and YZ designed, conceived the study, developed the project, and revised the manuscript. HL, JZ, YW, and LZ performed the experiments and analyzed the data. All authors contributed to the scientific discussions and manuscript preparation.

## Funding

This research was supported by the National Key Research and Development Program of China (2021YFC2600600), Beijing Natural Science Foundation (6234048), and the Basic Research Fund of the Chinese Academy of Inspection and Quarantine (2022JK09).

## Conflict of interest

YW is employed by Biowavelet Ltd.

The remaining authors declare that the research was conducted in the absence of any commercial or financial relationships that could be construed as a potential conflict of interest.

## Publisher’s note

All claims expressed in this article are solely those of the authors and do not necessarily represent those of their affiliated organizations, or those of the publisher, the editors and the reviewers. Any product that may be evaluated in this article, or claim that may be made by its manufacturer, is not guaranteed or endorsed by the publisher.
